# Epidemiological Study on the Dose–Effect Association between Physical Activity Volume and Body Composition of the Elderly in China

**DOI:** 10.3390/ijerph17176365

**Published:** 2020-09-01

**Authors:** Xiao Hou, Zheng-Yan Tang, Yu Liu, Yu-Jie Liu, Jing-Min Liu

**Affiliations:** 1 Department of Sports Science and Physical Education, Tsinghua University, Beijing 100084, China; houxiao18@mails.tsinghua.edu.cn (X.H.); tangzy19@mails.tsinghua.edu.cn (Z.-Y.T.); liu-yj17@mails.tsinghua.edu.cn (Y.-J.L.); 2 Jurong Center for Disease Control and Prevention, Jurong 212400, China; asialiuyu@163.com

**Keywords:** aging, old adults, physical activity, body composition, IPAQ

## Abstract

Purpose: The purpose of this study was to investigate Chinese old adults’ different body compositions in response to various levels of physical activity (PA). To the best of our knowledge, this is the first study to demonstrate the dose–response relationship between PA and body composition in old adults. Methods: 2664 participants older than 60 years (males: *n* = 984, females: *n* = 1680) were recruited for this cross-sectional health survey. PA was assessed by the short form of the International Physical Activity Questionnaire (IPAQ), and the body composition was measured by bioelectrical impedance analysis (BIA) instruments. The differences of separate body composition indices (lean body mass, LBM; bone mass, BM; and fat mass, FM) of older participants with different PA levels (below PA recommendation and over PA recommendation) were examined using the one-way analysis of variance (ANOVA). To compare the differences of three body composition indices with six different multiples of PA recommendation (0–1 REC, 1–2 REC, 2–4 REC, 4–6 REC, >6 REC), the one-way ANOVA and Turkey’s test was used for the post hoc analysis to identify the upper PA-benefit threshold in different indices of body composition. Results: The LBM and BM are significantly higher and the FM are significantly lower in old adults performing more PA volume than the WHO recommendation, compared with individuals performing less PA volume than the WHO recommendation. There were significant increases in LBM for males in “1–2 REC”, “2–4 REC”, and “>6 REC” groups, compared with the “0–1 REC” group; and there were significant increases in BM for males in “1–2 REC”, “2–4 REC”, compared with the “0–1 REC” group. The best PA volume for LBM and BM in females was the PA volume of “2–4 REC”. Additionally, whether males or females, there was no significant difference in FM between the “0–1 REC” group and other separate groups. Conclusion: The PA volume that causes best benefit for body composition of the elderly occurs at 1 to 2 times the recommended minimum PA for males, while it occurs at 2 to 4 times that recommended for females. No additional harms for old adults’ body composition occurs at six or more times the recommended minimum PA.

## 1. Introduction

In the last century, people’s life expectancy has increased dramatically, leading to huge changes in the population structure of the world [1]. Aging has become a global phenomenon, affecting a variety of countries around the world, especially China, which has a large population base [2]. It is predicted that by 2050, 20% of the world’s population (about 2 billion people) will be over 60 years old, of which 400 million people will be over 80 years old [3]. The unhealthy state of advanced ages will not only negatively affect their activities of daily living (ADL) and health-related quality of life (HRQoL), but also result in heavy economic, political, and social burdens on families and countries [4].

The potential changes of body composition may be one of the most common determinants of frailty and unhealthy state in old ages [5]. Several studies have confirmed that the elderly with more muscle mass and less muscle fat infiltration have better physical function and lower mortality [6,7,8,9,10]. Adam et al. assessed 1803 old adults’ five-year changes of body composition using computed tomography and dual x-ray absorptiometry and showed that losing thigh muscle area was associated with higher mortality and losing intermuscular thigh fat was protective against mortality [10]. Sarcopenia or the age-related loss of skeletal muscle mass can also increase the risk of falling and cause old adults’ frailty [11,12]. In terms of fat composition, it has been proved that the increase in fat content in the elderly is related to the occurrence of metabolic syndrome, a disorder disease including dyslipidemia, insulin resistance, and hypertension [13,14]. Several studies have demonstrated that overweight and obesity could aggravate arterial stiffness through increasing blood pressure [15,16,17] or metabolic disturbances [18], which could directly result in the occurrence of cardiovascular diseases in the elderly. Osteoporosis due to the loss of bone mass is also a common unhealthy state in old adults, and it is highly related to the risk of fractures [19]. There are lots of poor post-fracture outcomes, such as mortality, functional decline, and loss of quality of life [20,21]. For example, with a hip fracture after 12 months, more than one-third of patients less than 80 years old and almost 56% of patients older than 80 years old are unable to walk independently [22]. 

Several studies have proved that there is a significant correlation between physical activity (PA) and old adults’ healthy state. It has been consistently believed that regular PA is the most effective non-medicine intervention to improve old adults’ ability to carry out activities of daily living [23], muscular strength [24], balance [25], and to reduce the risk of falls [26], disability [27], cardiovascular disease [28], stroke [29], diabetes [30], and several cancers [31]. These positive effects of PA on functional health, to some extent, may attribute to the improvement of body composition. For example, Minoru et al. designed a cluster-randomized controlled trial and found that the six-month walking intervention based on pedometer could effectively increase the skeletal muscle mass index and hence prevent the sarcopenia in older adults, particularly in those who are frail [32]. Although body composition may mediate the effect of PA on health, few studies investigated the accurate dose–response relationship between PA and separate indices of body composition in the elderly. 

World Health Organization recommended old adults aged 65 years old and above should do at least 150 minutes of moderate-intensity PA (MPA) or 75 minutes of vigorous-intensity PA (VPA) throughout the week (at least 10 minutes each), or an equivalent combination (600 metabolic equivalent minutes per week) of moderate- and vigorous-intensity PA (MVPA) [33]. However, the guidelines did not define the specific benefits and the upper threshold of benefits for old adults’ PA, and did not note whether there were potential harms associated with very high levels of PA. 

To the best of our knowledge, this is the first study to demonstrate the dose–response relationship between PA and body composition in old adults. In this study, (1) we quantified the PA volume that could cause the best benefits for separate indices of body composition (e.g., lean body mass, LBM; bone mass, BM; and fat mass, FM) and the trend for different body compositions benefited from PA for old adults; (2) and we evaluated and explained whether there were excess harms for old adults’ body composition when conducting as much PA as possible.

## 2. Materials and Methods 

### 2.1. Study Population

A cross-sectional health survey design was used in this study. A total of 2664 elderly subjects older than 60 years were recruited through a large information campaign from Jurong Center for Disease Control and Prevention in Jiangsu Province, China. Among these samples, there were 984 males (median age: 67 years; range: 60–90 years) and 1680 females (median age: 65 years; range: 60–91 years). Inclusion criteria were as follows: over 60 years of age, absence of contraindication for conducting body composition measurement by bioelectrical impedance analysis (BIA) instruments, absence of clinically significant cardiovascular disease and severe acute or terminal illness, absence of physical disability that hinders PA, capable and willing to provide informed consent. 

### 2.2. Measurements and Instruments 

The recorded sociodemographic information in this survey included age, smoking habits (never, yes every day, yes but not every day), educational level (never educated, primary school, junior high school, senior high school, college), marital status (married, single, bereft of one’s spouse), occupation (farmer, worker, unemployed, retire, mental worker, other). See Table 1. The outcome included PA based on questionnaire, and separate indices of body composition (LBM, BM and FM) based on BIA. All measurements were conducted on the same day for each subject.

PA was assessed by the short form of the International Physical Activity Questionnaire (IPAQ). In this study, IPAQ was used to collect the volume of MPA and VPA per week based on the metabolic rate (MET) energy expenditure estimates for the last 7 days. The volume of PA (MET × min/wk) was calculated by multiplying the metabolic rate (MET) and the minutes spent on that activity per week (min/wk). In IPAQ, the MET values and formulas for computation of MET × min/week were derived from a variety of work undertaken during IPAQ reliability studies [34]. The IPAQ has acceptable measurement properties and has been used in several researches for older people [1,3]. Additionally, the Chinese version of IPAQ questionnaire had been proved to have high validity and reliability for assessing PA in Chinese old adults and it was an acceptable instrument for generating internationally comparable data on PA in this population [35]. Four formulas of physical activity in IPAQ were defined:

Walking MET × min/wk = 3.3 × walking minutes × walking days(1)

MPA MET × min/wk = 4.0 × MPA minutes × moderate days(2)

VPA MET × min/wk = 8.0 × VPA minutes × vigorous-intensity days(3)

Total PA MET × min/wk = sum of Walking + MPA + VPA MET × minutes/week scores(4)

According to Formulas (2) and (3) of PA volume based on the IPAQ, we calculated the MPA and VPA volume (MET × min/wk) during the last week respectively for each participant, and added up the “MET × min/week” of these two categories of PA to get the total “MET × min/week” of MVPA in the last week. Then, based on the WHO recommended minimum MVPA volume (600 MET × min/wk), we grouped the elderly participants into five different multiples of this recommendation (0–1 REC, <600 MET × min/wk; 1–2 REC, 600–1200 MET × min/wk; 2–4 REC, 1200–2400 MET × min/wk; 4–6 REC, 2400–3600 MET × min/wk; >6 REC, >3600 MET × min/wk). The participants who performed more than 600 MET × min/wk of MVPA volume met and exceeded the minimum recommendation, and others were those participants whose MVPA volume was below the WHO recommendation.

To measure the body composition, the BCAII (Body Composition Analyzer II, TFHT, Beijing, China) tetrapolar eight-point tactile electrode system was used. This device was based on BIA technique and was an accessible, inexpensive method for estimating total body water, extracellular water, fat-free mass, and body cell mass. With these estimated parameters, this method could allow the quantitative assessment of different body components in the human body [36]. The BCAII device had been applied in many scientific researches, and the measurement and details of BCAII had been published by several researchers [37,38,39]. Using this device, we assessed a series of quantitative values of body composition indicators including lean body mass (LBM), bone mass (BM), and fat mass (FM) in this study. Considering individual differences in body mass, the body composition variables (LBM, BM, FM) were presented and analyzed relative to body weight. The LBM was calculated by dividing an individual’s LBM in kilograms by the body weight in kilograms (unit: kg/kg). The BM was calculated by dividing an individual’s BM in grams by the body weight in kilograms (unit: g/kg). The FM was calculated by dividing an individual’s FM in kilograms by the body weight in kilograms (unit: kg/kg). In addition, the LBM and FM are presented as the percentage of body weight (%) in Table 2 and Table 3.

### 2.3. Procedures

When older subjects agreed to participate in this study, they received a questionnaire of specific information about illness, physical state, or disability to verify whether they met the inclusion criteria of this study. After providing information about the study (purpose, expected time and procedure of the questionnaire interview and body composition assessment), an informed consent was signed. All elderly participants in this study completed all measurement protocols on one day.

The interviewer read each question of IPAQ loudly to ensure that the subjects could understand the meaning of each item well and then wrote down the old subject’s answer in the questionnaire. After the survey of PA, the subject stood on the footboard of the BCAII device without shoes, metal accessories and in lightweight clothing, and held two handles of BCAII with two hands to measure the body compositions. All BIA data was collected at the similar times of day (from 7:00 a.m. to 9:00 a.m.). All subjects were fasted and restricted from fluid consumption after getting up, and also the vigorous exercise was prohibited. 

### 2.4. Ethical Considerations

All participants had detailed procedures introduced to them before participating in the study and then signed the informed consent documents. Participants were also clearly informed that they could withdraw from the study at any time for any reason. Any participant’s own PA and body composition measurement results in this study could be made into one’s health report and then provided to each participant. The authors declared that all the experiments of this study complied with the current laws of China in which they were performed. The study was approved by the medical ethics committee of Tsinghua University. 

### 2.5. Statistical Analysis

Results were expressed as Mean ± SD for continuous variables or frequencies (percentages) for categorical variables. We also reported the 95% confidence interval (95% CI) and variable range for the results. The subgroup analysis according to gender was performed in this study. The difference of separate body composition indicators (LBM, BM, FM) of older participants with different PA levels (below PA recommendation and over PA recommendation) were examined using the one-way analysis of variance (ANOVA). To compare the difference of these three body composition indicators with five different multiples of PA recommendation (0–1 REC, 1–2 REC, 2–4 REC, 4–6 REC, >6 REC), the one-way ANOVA and Turkey’s test was used for the post hoc analysis to identify the upper PA-benefit threshold in different body composition indices. The level of the significance was set at *p* < 0.05. In this study, we also indicated the significance level at *p* < 0.01. The statistical analyses were implemented by using the SPSS (Version 22, Chicago, IL, USA). 

## 3. Results

### 3.1. Demographic Analysis

The descriptive characteristics and PA level of 2664 participants are shown in Table 1. The majority of old adults performed 2–4 times the recommended volume of physical activity, 75.2% of the participants in this survey could meet the WHO minimum recommended PA volume. Only a small minority of old adults performed more than 6 times the PA recommendation. 

### 3.2. The Difference of Separate Body Composition of Old Adults with Different Physical Activity Level

Table 2 shows the difference in mean values of LBM, BM, and FM of participants with different PA levels among different gender groups. For the LBM, whether males or females, the participants performing PA volume over PA recommendation (male: 72.9 ± 9.4, female: 64.3 ± 7.9) had an LBM significantly higher than those performing PA volume below PA recommendation (male: 69.1 ± 6.1, female: 62.6 ± 9.0, *p* < 0.01). For the BM, the male participants performing PA volume over PA recommendation (49.2 ± 5.7) had a BM significantly higher than those performing PA volume below PA recommendation (45.1 ± 5.4, *p* < 0.05), and similar results presented in the female participants (44.6 ± 5.0 vs. 41.2 ± 5.9, *p* < 0.01). For the FM, there was also a significant difference between participants performing PA volume over PA recommendation and those performing PA volume below PA recommendation (male: 25.8 ± 8.1 vs. 27.3 ± 6.7, female: 33.0 ± 6.7 vs. 35.4 ± 17.6, *p* < 0.05).

### 3.3. The Dose–Effect Association between Physical Activity Volume and Body Composition of Old Adults

The LBM, BM, and FM of the old participants with five different multiples of PA recommendation are shown in Table 3. Using multi-level one-way ANOVA analysis, we could find that there were significant differences in LBM and BM between different multiples of PA recommendation groups in males or females. Additionally, after the Turkey’s test post hoc analysis, we could distinguish the specific significant differences from these five groups.

For the LBM, the highest mean value of LBM in males occurred in the “>6 REC” group (73.4 ± 8.9), which was significantly higher than that in the “0–1 REC” group (69.1 ± 6.1, *p* < 0.05). However, the LBM in the “1–2 REC” group (73.3 ± 9.1) and the “2–4 REC” group (73.0 ± 3.9) was also significantly higher than that in “0–1 REC” group (*p* < 0.05). For females, the highest mean value of LBM occurred in the “2–4 REC” group (66.4 ± 8.3), which was significantly higher than that in the “0–1 REC” group (62.6 ± 9.0, *p* < 0.05). 

For the BM, the highest mean value of BM in males occurred in the “1–2 REC” group (50.0 ± 5.4), which was significantly higher than that in the “0–1 REC” group (45.1 ± 5.4, *p* < 0.05). Furthermore, the BM in the “2–4 REC” group (48.6 ± 5.9) was also significantly higher than that in “0–1 REC” group (*p* < 0.05). For females, the highest mean value of BM occurred in the 2–4 REC group (45.9 ± 4.6), which was significantly higher than that in the “0–1 REC” group (41.2 ± 5.9, *p* < 0.05). 

For the FM, although the mean value of FM in “0–1 REC” group, whether in males or females, was higher than that in other groups, there was no significant difference between “0–1 REC” group and other separate groups after using post hoc analysis. 

## 4. Discussion

To the best of our knowledge, our findings on the elderly’s PA–body composition dose–response curve offer two unique and important contributions to the old adults’ non-medical health care and guidelines: (1) Old people of different genders have a different PA benefit threshold on different indicators of body composition. For old males, the best benefits for the LBM will occur when they perform a PA volume more than six-fold the PA recommendation suggested by WHO (>6 REC); and the maximum BM benefit of PA appears to be probably one to two times the WHO recommended minimum PA (1–2 REC). For old females, the best PA volume that could cause the maximum benefits for the LBM and BM appears to be probably two to four times WHO recommended minimum PA (2–4 REC). (2) There does not appear to be a decreased LBM and BM risk when the PA exceed six times WHO recommended minimum PA, compared with the PA volume below WHO recommended minimum PA. Although there was no significant difference in FM between five groups with different multiples of PA recommendation, there was a downward trend in FM with the increase in PA levels. Hence, it indicated that there were no additional harms for old adults’ body composition when conducting as much PA as possible. 

The descriptive statistical results of the subjects’ sociodemographic factors and PA in this study indicated that most old participants conducted 2–4 times WHO recommended PA volume and more than 75% Chinese old participants in our study meet the standard of WHO recommended PA volume for the elderly, and the rate to reach the standard was relatively high all over the world. An investigation in “Lancet” [40] revealed the proportion of inactive adults aged 60 years or older in Southeast Asia was approximately 30%, which was much lower than that of individuals of the same age in America, Europe, and some other counties. It implied that the Chinese old adults participating in our study were even more active than old adults in Southeast Asia, who might be the most active old adults all over the world, according to a previous scientific report. This may be because that the majority of old participants in our study were from Jurong city, an urban–rural junction area in China. Most of the old adults there were farmers, and the low-income farmers might do more PA volume and do MVPA more frequently (include occupational PA) [41] than those with higher socioeconomic status [42]. We also found that, among all sociodemographic factors in this study, educational level was the most important influential factor for old adults’ PA. The percentage of inactive old participants was significantly higher in individuals who were never educated (26.1%) than in those who attended primary school (24.9%), junior high school (21.8%), senior high school (19.9%), and college (0%). With the increase in educational level, the rate to reach WHO recommended minimum PA was getting higher. Previous studies about the association between education and PA provided inconsistent results. Our findings seemed to support Kwasniewska’s results that the likelihood of physical inactivity was higher in less educated individuals (*p* < 0.01) [43]. However, Luis et al. demonstrated that people with higher educated levels spent significantly (*p* < 0.001) more time sitting than those with low education levels [44]. This may be because that educational level has different effects on different categories of PA. For example, one study revealed that education was positively correlated to MVPA in transportation time and in leisure time, whereas it was negatively associated with the overall MVPA and the MVPA during working hours [45]. This reminds us that our research has limitations, because we did not distinguish the types of MVPA. In further study, we can investigate the PA volume using IPAQ (long term), which include five different PA domains (job-related PA, transportation PA, housework-related PA, recreation-related PA, and sitting).

The results about the body composition of participants with different PA volumes showed that the PA volume over the WHO PA recommendation would significantly produce more benefits in BM, LBM, and FM. Specifically, we observed that the percentage of LBM in males whose PA volume reached and exceeded the WHO recommended minimum PA (72.9%) was 3.8 percentage points higher than that in males whose PA volume did not reach the WHO recommended minimum PA (69.1%). Compared with females whose PA volume did not reach the WHO recommendation (62.6%), LBM was 1.7 percentage points higher in females whose PA volume exceeded the WHO recommendation (64.3%). Similarly, there was also a 4.1 g/kg higher BM in males performing below PA recommendation than in males performing over PA recommendation, and a 3.4 g/kg higher BM for females. For the percentage of body fat [8], PA could also cause a significant decline in males (25.8% vs. 27.3%) and females (33.0% vs. 35.4%). A variety of studies has proved that PA was an additional significant contributor for LBM [5], BM [46], and FM [47,48] in both men [49] and women [50], especially for the elderly. It was demonstrated that keeping physically active during aging could slow down the decreasing and changes in skeletal muscle tissue induced by aging [51]. Bielemann et al. believed that in the whole life, PA was very important for BM. They also explained that due to the low participation in peak strain activities, a lower association was found in females [52]. Although PA significantly increased the BM in both males and females in our study, the benefit of PA for BM in males was exactly more than that in females. Furthermore, it was reported that LBM could increase BM due to the physical loading reasons [53]. To some extent, old females could improve the BM through doing more high-load PA, which could strengthen muscles and LBM. Performing more PA volume than the WHO recommended minimum PA could significantly reduce the FM in both old males and females. It indicated that those individuals who were more active had a lower body fat percentage [47].

We also found that a different body composition mass had a different physical activity benefit threshold in different genders, through dividing the participants into five different multiples of WHO PA recommendation. The best benefit in LBM occured among males performing more than six times the recommended minimum PA and females performing 2 to 4 times the recommended minimum PA. While the PA volume of “1–2 REC” and “2–4 REC” also produced remarkable benefit for the LBM in males, after post hoc analysis, no significant difference was found in“1–2 REC”, “2–4 REC”, and “>6 REC”. Therefore, we suggest the PA volume of “1–2 REC” as the best choice for old males to acquire extra benefit, compared with the PA volume of “0–1 REC”. The best benefit in BM occurs among males performing 1 to 2 the recommended minimum PA and females performing 2 to 4 times the recommended minimum PA. Furthermore, for males, the PA volume of “2–4 REC” can cause extra benefit, compared with the PA volume of “0–1 REC”. But the BM of “1–2 REC” is significantly higher than that of “2–4 REC” after post hoc analysis. We found that the best benefit threshold of PA volume for LBM and BM is different in males and females, the PA volume that can produce best benefit for females is more than that for males. This may be because nearly all old female adults aged over 60 years are menopausal women, and due to menopause, there is a loss in the main female sex steroid hormone (i.e., Estradiol) [54,55], which has been proved to have an impact on decreasing muscle mass [56,57] and bone mass [58]. Hence, compared with men, women need more accumulation of PA to generate benefits in LBM and LBM. In terms of FM, the PA volume in four different multiples of REC groups that exceeds the recommended minimum PA does not seem to significantly reduce the FM of the elderly in our study. The possible reason might be that the intensity of PA we investigated and calculated in this study was moderate and vigorous intensity. In our study, we only calculated the MVPA to match the WHO recommendation for the elderly. However, the decrease in body fat is mainly due to the relative low-intensity aerobic training and endurance exercises and the increase in lean mass is mainly caused by relative high-intensity resistance training [59].We did not calculate the walking MET *min/wk, which might be the most common type of PA for old people, and this type of aerobic PA could exert beneficial impact on FM. This also implies that the WHO PA recommendation for the elderly has shortcomings. The long-duration and low-intensity aerobic PA is also meaningful for the body composition and health of the elderly. In the future, PA recommendation guidelines should contain different intensities of PA, even the low-intensity PA. 

It is worth mentioning that we observed an increased benefit for LBM and BM in individuals performing six or more times the recommended minimum PA, compared with that in participants performing less PA than the recommended minimum PA. It meant that there was no additional harms and risks for old adults’ body composition when conducting as much PA as possible. 

Our study has several limitations that need to be addressed. First, this study is limited by its cross-sectional design, it precludes any conclusions on causality. Future studies may perform a longitudinal study design to confirm our results. Second, this study estimated the volume of physical activity using IPAQ, and it requires participants to self-report and recall their physical activity of the last week, which can produce recall bias. Some studies have indicated the IPAQ could overestimate total physical activity [60,61]. Third, our study only evaluated the effect of total MVPA volume on different body composition indices of the elderly, but did not consider the effect of different PA domains on the three indices of body compositions and the effect of low-intensity PA on FM. In the next study, we will focus more on this problem.

## 5. Conclusions

Performing more PA volume than the WHO recommended minimum PA could generate more benefit in BM, LBM, and FM in the elderly. The PA volume that causes the best benefit for body composition of the elderly occurs at 1 to 2 times the recommended minimum PA for males, while it occurs at 2 to 4 times that recommended for females. No additional harms for old adults’ body composition occurs at six or more times the recommended minimum PA. These findings will be useful for updating PA guidelines for Chinese old adults based on the body composition. 

## Figures and Tables

**Table 1:** Descriptive characteristics and physical activity of 2664 participants.

Characteristic	Participants	PA MET × min/wk No. (%) ^a^				
		0–600 (0–1 REC)	600–1200 (1–2 REC)	1200–2400 (2–4 REC)	2400–3600 (4–6 REC)	>3600 (>6 REC)
**participants**	2664	660 (24.8)	630 (23.6)	862 (32.3)	304 (11.4)	208 (7.8)
**age(years)**						
60–69 (include 69)	1914	469 (24.5)	463 (24.2)	620 (32.4)	214 (11.2)	148 (7.7)
70–79 (include 79)	626	166 (26.5)	141 (22.5)	193 (30.8)	77 (12.3)	49 (7.9)
≥80	124	25 (20.2)	26 (21.0)	49 (39.5)	13 (10.5)	11 (8.8)
**gender**						
male	984	241 (24.5)	224 (22.8)	320 (32.5)	113 (11.5)	86 (8.7)
female	1680	419 (24.9)	406 (24.2)	542 (32.3)	191 (11.4)	122 (7.3)
**smoking**						
never	2134	530 (24.8)	500 (23.4)	695 (32.6)	250 (11.7)	159 (7.4)
yes, every day	485	120 (24.7)	117 (24.1)	153 (31.5)	48 (9.9)	47 (9.7)
yes, but not everyday	45	10 (22.2)	13 (28.9)	14 (31.1)	6 (13.3)	2 (4.4)
**educational level**						
never educated	1664	435 (26.1)	403 (24.2)	518 (31.1)	181 (10.9)	127 (7.6)
primary school	414	103 (24.9)	86 (20.8)	139 (33.6)	46 (11.1)	40 (9.7)
junior high school	422	92 (21.8)	97 (23.0)	146 (34.6)	50 (11.8)	37 (8.8)
senior high school	151	30 (19.9)	39 (25.8)	53 (35.1)	25 (16.6)	4 (2.7)
college	13	0 (0.0)	5 (38.5)	6 (46.2)	2 (15.4)	0 (0.0)
**marital status**						
married	2130	528 (24.8)	504 (23.7)	687 (32.3)	242 (11.4)	169 (7.9)
single	47	14 (29.8)	10 (21.3)	13 (27.7)	5 (10.6)	5 (10.7)
bereft of one’s spouse	487	118 (24.2)	116 (23.8)	162 (33.3)	57 (11.7)	34 (7.0)
**occupation**						
farmer	1828	464 (25.4)	446 (24.4)	557 (30.5)	209 (11.4)	152 (8.3)
worker	118	17 (14.4)	29 (24.6)	45 (38.1)	12 (10.2)	15 (12.7)
unemployed	420	119 (28.3)	90 (21.4)	144 (34.4)	42 (10.0)	25 (5.9)
retiree	231	45 (19.5)	54 (23.4)	91 (39.4)	29 (12.6)	12 (5.2)
mental worker	47	9 (19.1)	9 (19.1)	19 (40.4)	6 (12.8)	4 (8.5)
other	13	4 (30.8)	1 (7.7)	4 (30.8)	4 (30.8)	0 (0.0)

Abbreviations: PA: physical activity; MET: metabolic equivalent; min: minute; wk: week; REC: the WHO recommended physical activity minimum in old adults; ^a^ Frequencies are row percentage.

**Table 2:** The separate body composition indicators of participants with different physical levels.

Body Composition Indicators	Gender	Below PA Recommendation			Over PA Recommendation		
		M ± SD	95% CI	Range	M ± SD	95% CI	Range
LBM (%)	Male	69.1 ± 6.1	68.6–69.7	44.3–89.0	72.9 ± 9.4 **	72.3–73.6	48.4–86.7
	Female	62.6 ±9.0	63.0–62.2	39.7–78.1	64.3 ± 7.9 **	63.8–64.7	43.1–80.6
BM (g/kg)	Male	45.1 ± 5.4	44.7–45.6	23.0–71.4	49.2 ±5.7 *	48.8–49.6	28.4–89.7
	Female	41.2 ± 5.9	40.9–41.4	19.1–74.7	44.6 ± 5.0 **	44.3–44.8	20.9–71.3
FM (%)	Male	27.3 ± 6.7	26.7–28.0	9.7–55.2	25.8 ± 8.1 *	25.3–26.4	12.9–52.5
	Female	35.4 ± 17.6	34.9–35.8	22.3–60.0	33.0 ± 6.7 *	32.6–33.4	19.8–57.4

Abbreviations: PA, physical activity; LBM, lean body mass; BM, bone mass; FM, fat mass; M ± SD, mean ± standard deviation; CI, confidence interval; * Significant difference on separate body composition indices between different PA levels (*p* < 0.05); ** Significant difference on separate body composition indices between different PA levels (*p* < 0.01). “Below PA Recommendation” in the table represents “the PA volume performed by subjects is more than the WHO recommended PA volume”; “Over PA Recommendation” in the table represents “the PA volume performed by subjects is less than the WHO recommended PA volume”.

**Table 3:** The LBM, BM, and FM of participants with different multiples of PA recommendation.

Gender	Body Composition Indicators	0–1 REC	1–2 REC	2–4 REC	4–6 REC	>6 REC	*p*-Value	F	Eta^2^
Male	LBM (%)	69.1 ± 6.1	73.3 ± 9.1 *	73.0 ± 3.9 *	72.1 ± 4.0	73.4 ± 8.9 *	0.047	2.423	0.102
	BM (g/kg)	45.1 ± 5.4	50.0 ± 5.4 *^#^	48.6 ± 5.9 *	47.6 ± 4.7	49.4 ± 7.2	0.046	2.433	0.102
	FM (%)	27.3 ± 6.7	26.4 ± 4.7	25.7 ± 8.3	25.0 ± 8.8	25.2 ± 7.8	0.537	0.797	0.013
Female	LBM (%)	62.6 ±9.0	63.8 ± 7.1	66.4 ± 8.3 *	64.0 ± 11.3	64.8 ± 5.2	0.023	2.847	0.117
	BM (g/kg)	41.2 ± 5.9	43.4 ± 5.5	45.9 ± 4.6 *	43.0 ± 4.8	43.5 ± 3.7	0.045	2.443	0.105
	FM (%)	35.4 ± 17.6	33.4 ± 12.4	33.5 ± 11.7	32.2 ± 7.9	32.1 ±5.5	0.323	1.169	0.009

Abbreviations: LBM, lean body mass; BM, bone mass; FM, fat mass; REC, the WHO recommended physical activity minimum in old adults; * Compared with 0–1 REC PA volume, significant difference on separate body composition indices (*p* < 0.05); ^#^ Compared with 2–4 REC PA volume, significant difference on BM (*p* < 0.05); Eta^2^: Eta-squared, represent the effect size.
